# Refining the assessment of BI-RADS 4 lesions on breast magnetic resonance imaging

**DOI:** 10.1590/0100-3984.2025.0035-en

**Published:** 2025-12-15

**Authors:** Luciana Graziano, Bianca Miranda Lago, Camila Souza Guatelli, Juliana Alves, Mariah Carneiro Wanderley, Vinicius Cardona Felipe, Almir Galvão Vieira Bitencourt

**Affiliations:** 1 A.C.Camargo Cancer Center, São Paulo, SP, Brazil

**Keywords:** Breast neoplasms/diagnostic imaging, Radiology information systems/standards, Magnetic resonance imaging., Neoplasias da mama/diagnóstico por imagem, Sistemas de informação em radiologia/normas, Ressonância magnética.

## Abstract

**Objective:**

To evaluate the positive predictive value (PPV) of the imaging characteristics of breast lesions classified as BI-RADS category 4 (risk of malignancy > 2% to < 95%) on magnetic resonance imaging (MRI), in order to create an algorithm to subcategorize such lesions.

**Materials and Methods:**

This was a retrospective study including 199 breast lesions (131 nodules and 68 non-mass lesions) classified as BI-RADS 4 on MRI. Of the 199 lesions, 93 were excluded, for various reasons: they were lymph nodes; they were not biopsied or were not followed at our center; they were additional findings in patients with an established diagnosis of malignancy who underwent mastectomy without further investigation; or they were identified in examinations that were not recovered from the digital archive. Multivariate analysis was performed to identify the most relevant descriptors to predict malignancy and to build an algorithm to subcategorize lesions into BI-RADS 4A, 4B, and 4C. Four breast radiologists then tested the algorithm in another 95 patients with breast lesions classified as BI-RADS 4 on MRI, 27 (28.4%) of those lesions having previously been classified as malignant.

**Results:**

The descriptors statistically associated with malignancy in the multivariate analysis of the nodules were background parenchymal enhancement, margins, and the initial phase of the kinetic curve. An algorithm was developed by using these resources, and the PPV obtained for each category was 4.3% for BI-RADS 4A, 21.4% for BI-RADS 4B, and 78.9% for BI-RADS 4C. In the validation of the algorithm by the four breast radiologists, the PPV of the subcategories was within the BI-RADS malignancy ranges in almost all situations, the exceptions being the 11.1% that one evaluator obtained for category 4A and the 46.4% obtained for category 4C by another evaluator.

**Conclusion:**

The objective analysis employing the proposed algorithm proved useful for subdividing BI-RADS 4 mass lesions on MRI and showed better interobserver agreement than did the subjective analysis.

## Abstract (pt)

**Objetivo:**

Avaliar o valor preditivo positivo (VPP) das características de imagem de lesões mamárias classificadas na categoria BI-RADS 4 (risco de malignidade > 2% a < 95%) na ressonância magnética (RM), com a finalidade de criar um algoritmo para subcategorizar essas lesões.

**Materiais e Métodos:**

Estudo retrospectivo que incluiu 199 lesões (131 nódulos e 68 realces não nodulares) classificadas como categoria BI-RADS 4 pela RM das mamas. Foram excluídas 93 lesões por tratar-se de linfonodos, lesões não biopsiadas ou sem seguimento na instituição, achados adicionais na mama com diagnóstico estabelecido para malignidade e submetidas a mastectomia sem investigação adicional, e exames com falha no arquivo digital. A análise multivariada foi realizada para identificar os descritores mais relevantes para prever malignidade e construir um algoritmo para subcategorizar as lesões em 4A, 4B e 4C. Em seguida, o algoritmo foi testado por quatro radiologistas de mamas em outras 95 pacientes com lesões de mama classificadas como BI-RADS 4 na RM (27 lesões malignas; 28,4%).

**Resultados:**

Os descritores estatisticamente associados a malignidade na análise multivariada dos nódulos foram realce de fundo do parênquima, margens e fase inicial da curva cinética. Foi desenvolvido um algoritmo utilizando esses recursos e o VPP obtido para cada categoria foi de 4,3% para BI-RADS 4A, 21,4% para BI-RADS 4B e 78,9% para BI-RADS 4C. Na validação do algoritmo por quatro radiologistas mamários, o VPP das subcategorias estava nas faixas de malignidade especificadas pelo BI-RADS em quase todas as situações, exceto o de um observador que registrou VPP de 11,1% para categoria 4A do BI-RADS, e de outro observador que encontrou VPP de 46.4% para categoria 4C do BI-RADS.

**Conclusão:**

O algoritmo proposto foi útil na subdivisão de lesões de massa BI-RADS 4 na RM e mostrou melhor concordância entre os observadores do que a análise subjetiva.

## INTRODUCTION

Breast cancer is the most common malignant neoplasm in women worldwide. According to statistics published by the Brazilian National Cancer Institute, 73,000 cases of female breast cancer are expected to occur in Brazil in the 2023-2025 triennium^(1,2)^. That high incidence is related to multiple factors, including lifestyle changes, endocrine alterations, and reproductive history, as well as behavioral, environmental, and genetic factors^(3)^. The main strategy for reducing breast cancer mortality is early diagnosis. In this context, imaging methods are essential, allowing the identification of the disease in its early stages, when the chances of a cure are greater.

Breast imaging methods such as mammography, ultrasound, and magnetic resonance imaging (MRI) play a fundamental role in the identification and investigation of breast lesions, both in screening examinations for asymptomatic women and in diagnostic examinations for women with clinical complaints or alterations on previous examinations. The Breast Imaging Reporting and Data System (BI-RADS) of the American College of Radiology standardizes breast imaging reports, classifying findings to guide clinical management, facilitating the differentiation between benign and suspicious changes, thus ensuring better patient follow-up. However, the malignancy rate among biopsies of lesions classified as suspicious (BI-RADS category 4) is only 10-30%, meaning that most biopsies yield benign results, while causing unnecessary patient discomfort and anxiety, as well as increasing health care costs^(4)^.

Breast MRI is the most sensitive imaging method for diagnosing breast cancer. However, for lesions classified as suspicious and falling into BI-RADS category 4, the risk of malignancy is highly variable^(5)^ and the positive predictive value (PPV) of biopsied lesions based on MRI findings ranges from 20% to 60%^(6,7)^. For this reason, BI-RADS now suggests subdividing category 4-within which the risk of malignancy ranges from 2% to 95%-into three subcategories: 4A, in which the level of suspicion for malignancy is low (2-10% likelihood of malignancy); 4B, in which the level of suspicion for malignancy is intermediate (11-50% likelihood of malignancy), and 4C, in which the level of suspicion for malignancy is high (51-96% likelihood of malignancy). However, there are still no well-established criteria for this subdivision of BI-RADS category 4 in MRI.

The aim of this study was to evaluate the PPV of the imaging features of breast lesions classified in BI-RADS category 4 by MRI, with the aim of creating an algorithm to subcategorize these lesions into BI-RADS 4A, 4B, and 4C.

## MATERIALS AND METHODS

### Study design and population

This was a retrospective, single-center, observational study including patients who presented with lesions previously classified as BI-RADS category 4 and who subsequently underwent breast MRI. The study was approved by the local research ethics committee (Reference no. 71348217.8.0000.5432). From September 2016 to September 2017, a total of 2,700 breast MRI examinations were performed at our center. Among those examinations, there were 292 lesions that were classified as BI-RADS 4. Of those 292 lesions, 93 were excluded: 31 because they were axillary lymph nodes with suspicious findings; 18 because they were not biopsied and were not followed at our center; 13 because they were additional findings in patients with an established diagnosis of malignancy who did not undergo further investigation; and 31 because the images were not recovered from the digital archive. The final sample therefore comprised 199 lesions classified as BI-RADS 4, in 166 patients (135 patients with one lesion, 29 patients with two lesions, and two patients with three lesions). The mean age of the patients was 49.8 ± 11.9 years (range, 26-82 years; median, 48 years). Forty-one patients (24.7%) had a family history of breast cancer, 26 (15.7%) had a personal history of breast cancer, and 52 (31.3%) had current (untreated) breast cancer.

In addition to the initial sample, a second, independent sample of patients (N = 95) was included in the study. These patients also had lesions previously classified as BI-RADS 4, which were evaluated by four other radiologists (with 2-12 years of experience in breast imaging) using the same inclusion and exclusion criteria described above. Analyses of those lesions were performed objectively, as described in the results section.

### Breast MRI protocol

Each MRI scan was acquired with the patient in the prone position in a 1.5-T scanner-Signa HDxt (GE Healthcare, Milwaukee, WI, USA) or Ingenia (Philips Healthcare, Best, the Netherlands)-with a dedicated breast coil. Images acquired before and after intravenous contrast administration began with a scout view, which allows localization of the spatial distribution of breast tissue and from which further sequences are planned. The following sequences were obtained:

- axial unenhanced three-dimensional T1-weighted gradient-echo sequence, with the parameters repetition time/echo time (TR/TE), 4.3/1.4 ms; flip angle, 12°; field of view, 320 × 320; matrix, 307 × 512; signal average, 1; and slice thickness, 2.5 mm
- sagittal unenhanced T2-weighted short-tau inversion recovery sequences of both breasts, with the parameters TR/TE, 4,500/97 ms; matrix, 384 × 512; and slice thickness, 4.0 mm
- axial unenhanced echo-planar diffusion-weighted array spatial sensitivity encoding technique sequence, with the parameters TR/TE, 4,000/94 ms; matrix, 192 × 192; signal average, 3; slice thickness, 3 mm; and distance factor, 20%, diffusion gradient sensitization being applied in two orthogonal directions, with b values of 0 and 750 s/mm^2^, respectively
- a dynamic examination, comprising five axial (unenhanced and contrast-enhanced) three-dimensional fat-suppressed T1-weighted gradient-echo sequences, with no time interval between them, the contrast agent being gadolinium-Gadovist (Bayer Schering Pharma AG, Berlin, Germany), or Dotarem (Guerbet, Roissy, France)-injected at 0.1 or 0.2 mL/kg of body weight, respectively, followed by a bolus injection of 20 mL of saline solution, the first image being obtained before contrast injection, the second being obtained 20 s after contrast injection, and the remaining images being obtained sequentially over the following minutes, with post-processed images obtained from the dynamic images after the end of the examination, the unenhanced images being subtracted from the contrast-enhanced images to enhance visualization of the enhancing structures, including the areas of enhancement to be analyzed
- sagittal contrast-enhanced three-dimensional T1-weighted gradient-echo sequences of both breasts, with high spatial resolution, 1-mm slice thickness, and fat saturation.

### Image analysis

Two radiologists with 10 and 11 years of experience in breast imaging, respectively, reviewed the images and classified the lesions according to the BI-RADS criteria. Each radiologist, working independently, reviewed the selected BI-RADS 4 lesions, without access to the original report, clinical data, images from previous studies, or pathology findings.

The breast composition (predominantly adipose, sparsely fibroglandular, heterogeneously fibroglandular, or extremely fibroglandular) was evaluated, as were the pattern of background parenchymal enhancement (minimal, mild, moderate, or marked), the type of lesion (mass or non-mass), and the type of kinetic curve (persistent, plateau, or washout). Nodules, defined as space-occupying lesions ≥ 5 mm, were evaluated for shape (round, oval, or irregular), margins (well-circumscribed, irregular, or spiculated), and enhancement (homogeneous, heterogeneous, peripheral, or with non-enhancing internal streaks). Non-mass lesions, defined as those that do not occupy a defined space, were evaluated for distribution (focal, linear/ductal, regional, segmental, or multifocal) and internal enhancement pattern (homogeneous, heterogeneous, clumped, or in clustered rings).

In an attempt to stratify the risk of each lesion, the evaluators performed a “subjective” subdivision of BI-RADS category 4 into the subcategories 4A, 4B, and 4C. The gold standard was taken to be the histological results from percutaneous or surgical biopsies of lesions with results consistent with the imaging findings. For patients in whom there were no biopsy results, clinical and imaging follow-up for at least two years was performed to demonstrate the stability of the findings.

### Statistical analysis

The data obtained were stored in a the Research Electronic Data Capture database (Vanderbilt University, Nashville, TN, USA), and the statistical analysis was performed with the IBM SPSS Statistics software package, version 20.0 (IBM Corporation, Armonk, NY, USA). Categorical variables are expressed as absolute and relative frequencies. For each of the variables, interobserver agreement was analyzed by calculating the kappa statistic. The kappa values were categorized as follows^(8)^: 0.01-0.20 = poor agreement; 0.21-0.40 = fair agreement; 0.41-0.60 = moderate agreement; 0.61-0.80 = substantial agreement; and 0.81-0.99 = almost perfect agreement.

To compare scalar variables between two groups, we used Student’s t-test or the nonparametric Mann-Whitney test, as appropriate. In cases of three or more groups, analysis of variance or the nonparametric Kruskal-Wallis test was used. Categorical variables were analyzed by using 2 × 2 and 2 × 3 tables, with statistical significance assessed by Pearson’s chi-square test with Yates’ correction or Fisher’s exact test, as indicated. Results with a type I error probability less than or equal to 5% (*p* ≤ 0.05) were considered statistically significant.

For multivariate analysis, binary logistic regression was performed, with the outcome (benign vs. malignant) being used as the dependent variable. In the regression model, variables with a value of *p* < 0.10 in the univariate analysis were included as predictors. The odds ratio was calculated for each variable, and results with a value of *p* < 0.05 were considered statistically significant. The results of the final regression model were used to develop an algorithm for objectively subclassifying lesions as BI-RADS 4A, 4B, or 4C, according to the probability of malignancy of the most significant findings. Finally, the second group of radiologists tested the proposed algorithm in the independent sample of 95 breast lesions also classified as BI-RADS 4 on MRI, 27 (28.4%) of which had previously been classified as malignant.

## RESULTS

Two independent samples were evaluated: the first, composed of 199 lesions (subjective analysis), evaluated by two radiologists; and the second, composed of 95 lesions (objective analysis), evaluated by four other radiologists, using the same inclusion and exclusion criteria described in the methods section.

Of the 199 lesions analyzed in the initial sample (subjective analysis), 131 were nodules and 68 were non-mass lesions. Of those 199 lesions, 185 were submitted to biopsy, the results of which indicated that 140 (75.6%) were benign and 45 (24.3%) were malignant (Table 1). The remaining 14 lesions (7.5%) were not investigated but remained stable for more than two years, implying benignity. The descriptors evaluated on MRI are described in Table 2, as is the level of interobserver agreement, which was good or excellent for most descriptors.

**Table 1:** Histological diagnosis of benign and malignant breast lesions.

Histological diagnosis	(N = 185)^*^
Benign lesions, n (%)	140 (75.7)
Fibroadenoma	27 (14.6)
Stromal fibrosis	23 (12.4)
Papilloma	22 (11.9)
Other findings^†^	67 (36.8)
Malignant lesions, n (%)	45 (24.3)
Invasive carcinoma of no special type	18 (9.7)
Ductal carcinoma *in situ*	14 (7.6)
Invasive lobular carcinoma	6 (3.2)
Papillary carcinoma	3 (1.6)
Tubular carcinoma	1 (0.5)
Adenoid cystic carcinoma	1 (0.5)
Malignant phyllodes tumor	1 (0.5)
Non-Hodgkin lymphoma	1 (0.5)

* 14 of the 199 lesions were not biopsied.

† Other benign lesions (n = 45); radial scar (n = 3); tubular adenoma (n = 2); pseudoangiomatous stromal hyperplasia (n = 9); atypical ductal hyperplasia confirmed at surgery (n = 5); atypical lobular hyperplasia (n = 1); complex sclerosing lesion (n = 1); and lobular neoplasia (n = 1).

**Table 2:** Breast MRI findings and interobserver variability.

MRI findings	Reviewer 1 (N = 199)	Reviewer 2 (N = 199)	k
Breast composition, n (%)			0.727
Predominantly adipose or sparse fibroglandular tissue	58 (29.1)	54 (27.1)	
Heterogeneously or extremely fibroglandular	141 (70.8)	145 (72.9)	
Background parenchymal enhancement, n (%)			0.693
Minimal/discreet	142 (71.4)	157 (78.9)	
Moderate/sharp	57 (28.6)	42 (21.1)	
Type of finding, n (%)			0.861
Nodule	131 (65.8)	141 (70.8)	
Non-mass enhancement	67 (34.2)	58 (29.1)	
Nodule	(n = 131)	(n = 141)	
Shape, n (%)			0.423
Oval	68 (51.9)	105 (74.4)	
Round	28 (21.3)	22 (15.6)	
Irregular	35 (26.7)	14 (9.9)	
Margins, n (%)			0.632
Circumscribed	61 (46.6)	73 (51.8)	
Non circumscribed	70 (53.4)	67 (48.2)	
Internal enhancement, n (%)			0.420
Homogeneous	35 (26.7)	38 (26.9)	
Heterogeneous	88 (61.2)	86 (61.0)	
Peripheral	5 (3.8)	13 (0.7)	
Non-captive internal beams	3 (2.3)	3 (2.1)	
Enhancement type, n (%)			0.517
Ascendant	58 (44.2)	44 (31.2)	
Plateau	57 (43.5)	76 (53.9)	
Washout	16 (12.2)	21 (14.9)	
Non mass enhancement	(n = 68)	(n = 58)	
Distribution, n (%)			0.606
Focal	29 (42.6)	22 (37.9)	
Linear or ductal	13 (19.1)	15 (25.8)	
Regional	4 (5.9)	5 (8.6)	
Segment	21 (14.9)	15 (25.8)	
Multiple areas	1 (1.4)	1(1.7)	
Internal enhancement, n (%)			0.613
Homogeneous	24 (35.3)	12 (20.7)	
Heterogeneous	38 (55.9)	35 (60.3)	
Clumped	5 (7.3)	8 (13.8)	
Clustered rings	1 (1.5)	2 (3.4)	
BI-RADS subcategory, n (%)	(N = 199)	(N = 199)	0.500
4A	85 (42.7)	86 (43.2)	
4B	53 (26.6)	58 (29.1)	
4C	61 (30.6)	65 (32.6)	

In the univariate analysis between the BI-RADS descriptors and the histological result, the following variables presented statistically significant associations with a higher risk of malignancy (Table 3): breast composition, background parenchymal enhancement, nodule morphology, nodule margins, and kinetic curve (early and late phases). For nodules, the multivariate analysis showed that only background parenchymal enhancement, margins, and kinetic curve (early phase) presented statistical significance; for non-mass lesions, only the distribution was shown to be associated with an increased risk of malignancy, albeit without statistical significance (Table 4).

**Table 3:** Distribution of BI-RADS descriptors of benign and malignant lesions (N = 199) with univariate statistical analysis.

Variable	Result		P^*^
	Benig (n = 154)	Malignant (n = 45)	
Breast composition, n (%)			0.045
Predominantly adipose or sparse fibroglandular tissue	39 (25.3)	19 (42.2)	
Heterogeneously or extremely fibroglandular	115 (74.7)	26 (57.8)	
Background parenchymal enhancement, n (%)			0.043
Minimal or discreet	104 (67.5)	38 (84.4)	
Moderate or severe	50 (32.5)	7 (15.6)	
Type of injury, n (%)			0.448
Nodule	104 (67.5)	27 (60.0)	
Non-mass enhancement	50 (32.5)	18 (40.0)	
Nodule	(n = 104)	(n = 27)	
Morphology, n (%)			< 0.0001
Oval or round	84 (80.8)	12 (44.4)	
Irregular	20 (19.2)	15 (55.6)	
Margins, n (%)			< 0.0001
Circumscribed	58 (55.8)	3 (11.1)	
Irregular or spiculated	46 (44.2)	24 (88.9)	
Enhancement pattern, n (%)			0.526
Homogeneous or non-capturing internal beams	32 (30.8)	6 (22.2)	
Heterogeneous or peripheral	72 (69.2)	21 (77.8)	
Curve - initial phase, n (%)			< 0.0001
Slow or moderate	111 (84.1)	12 (34.3)	
Rapid	21 (15.9)	23 (65.7)	
Curve - late phase, n (%)			< 0.0001
Progressive	68 (51.5)	3 (8.6)	
Plateau	56 (42.4)	23 (65.7)	
Washout	8 (6.1)	9 (25.7)	
Non-mass enhancement	(n = 50)	(n = 18)	
Distribution, n (%)			0.169
Focal or regional or multiple regions	28 (56.0)	6 (33.3)	
Linear or segmental	22 (44.0)	12 (66.7)	
Enhancement pattern, n (%)			0.999
Homogeneous	18 (36.0)	6 (33.3)	
Heterogeneous/clustered rings/clumped	32 (64.0)	12 (66.7)	
Additional findings, n (%)	(n = 154)	(n = 45)	0.544
No	142 (92.2)	40 (88.9)	
Yes	12 (7.8)	5 (11.1)	
Diameter on the longest axis (mm)			0.183^†^
Mean ± SD	17.91 (18.48)	24.09 (29.04)	
Median (range)	10.5 (3.0-123.0)	12.0 (3.0-150.0)	

* Chi-square test with continuity correction.

† Student’s t-test for independent samples.

**Table 4:** Results of the multivariate analysis to calculate the risk of malignancy of the most significant variables identified in the final logistic regression model.

Variable	Categories	Odds ratio	95% Cl	*P*
Breast enhancement pattern	Moderate or severe	0.206	0.054-0.790	0.021
Nodule: margins	Irregular or spiculated	7.457	1.876-29.632	0.004
Nodule: kinetic pattern	Initial phase - rapid	12.06	3.982-36.573	< 0.0001
Non-mass enhancement: distribution	Linear or segmental	2.545	0.824-7.863	0.104

**Figure 1:**
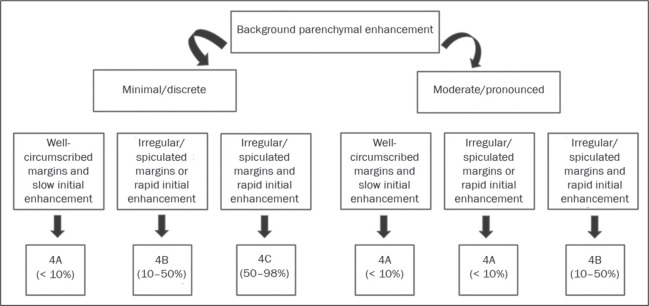
Proposed algorithm for objective classification of nodules in the BI-RADS subcategories 4A, 4B, and 4C.

**Figure 2:**
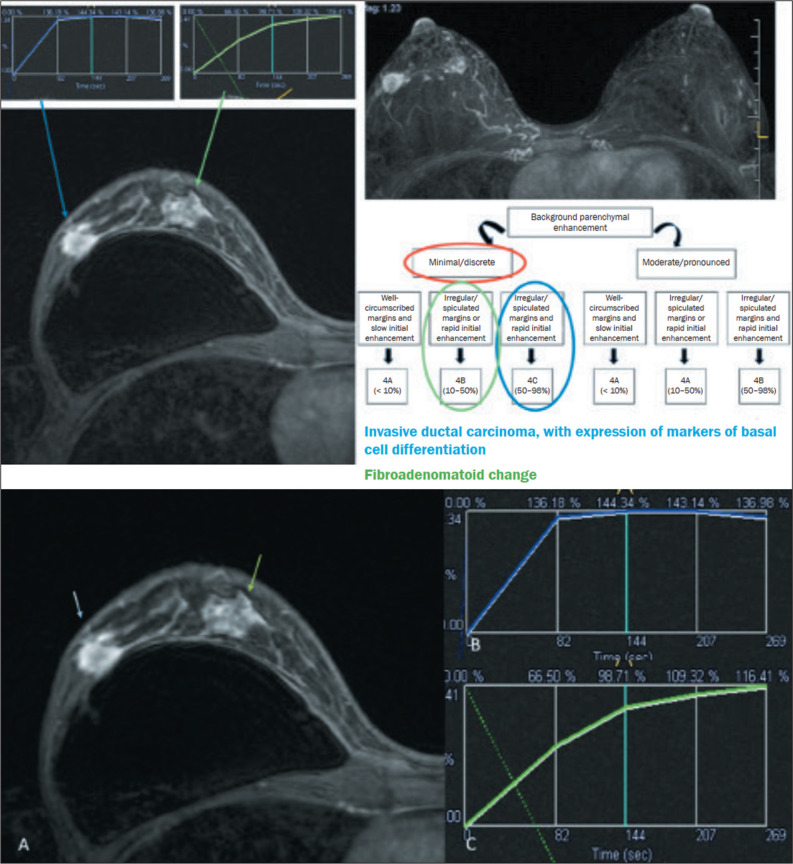
Example of breast MRI (A) showing two nodules in the right breast with irregular shape and margins, heterogeneous enhancement, and a kinetic curve of early initial enhancement for the nodule with the blue arrow (B), classified as BI-RADS 4C, and delayed enhancement for the nodule with the green arrow (C), classified as BI-RADS 4B. The histological findings were invasive breast carcinoma and fibroadenomatoid change, respectively.

The PPV for malignancy of nodules was also evaluated according to the variables that presented a statistically significant difference, including background parenchymal enhancement, nodule margins, and the pattern of nodular enhancement (early phase), as shown in Table 5. On the basis of those values, an algorithm was proposed for objectively organizing the lesions into BI-RADS subcategories 4A, 4B, and 4C (Figure 1). In the subjective analysis of the lesions in subcategories 4A (n = 60), 4B (n = 34), and 4C (n = 37), the PPV for malignancy was found to be 0%, 11.8%, and 62.2%, respectively, compared with the results obtained for the objective analysis (Table 6). Figure 2 illustrates cases evaluated with the proposed algorithm. It was not possible to perform this evaluation for non-mass lesions, because none of the variables evaluated demonstrated a statistically significant association with malignancy.

**Table 5:** Probability of malignancy of nodules according to significant characteristics in the multivariate analysis.

n	Breast enhancement pattern	Margins	Curve - initial phase	Probability of malignancy	95% Cl
29	Minimal/mild	Circumscribed	Slow/mode rate	3.35%	0.90-12.20%
5	Minimal/mild	Circumscribed	Rapid	29.50%	9.50-62.50%
30	Minimal/mild	Irregular/spiculated	Slow/mode rate	20.55%	10.30-36.80%
19	Minimal/mild	Irregular/spiculated	Rapid	75.73%	55.40-88.70%
22	Moderate/severe	Circumscribed	Slow/mode rate	0.71%	0.10-4.30%
5	Moderate/severe	Circumscribed	Rapid	7.94%	1.70-30.60%
14	Moderate/severe	Irregular/spiculated	Slow/mode rate	5.06%	1.30-18.00%
7	Moderate/severe	Irregular/spiculated	Rapid	39.13%	15.80-68.80%

**Table 6:** Histological results of the subjective and objective analyses of BI-RADS in nodules (n = 131).

BI-RADS for nodular enhancement	Benign n (%)	Malignant n (%)	Total n
Subjective analysis			
4 A	60 (100.0)	0 (0.0)	60
4 B	30 (88.2)	4(11.8)	34
4C	14 (37.8)	23 (62.2)	37
Objective analysis			
4 A	67 (95.7)	3 (4.3)	70
4 B	33 (78.6)	9 (21.4)	42
4C	4 (21.1)	15 (78.9)	19

In the objective analysis, the algorithm was tested by the four other breast radiologists, and the PPVs for the subcategories were found to be within the ranges specified by the BI-RADS in nearly all situations (Table 7). However, one observer reported a PPV of 11.1% for subcategory 4A and another reported a PPV of 46.4% for subcategory 4C. In addition, there was better agreement among the four radiologists who performed the objective analysis (using the algorithm) than between the two radiologists who performed the subjective analysis, with intraclass correlation coefficients of 0.473 and 0.292, respectively.

**Table 7:** PPV of the BI-RADS 4 subcategories assigned by four evaluators in the subjective analysis and in the objective analysis (using the algorithm proposed) in the independent sample (N = 95).

Analysis	BI-RADS	Evaluator 1 n/N (%)	Evaluator 2 n/N (%)	Evaluator 3 n/N (%)	Evaluator 4 n/N (%)	ICC
Subjective	4A	2/41 (4.9)	1/8 (12.5)	1/26 (3.8)	2/29 (6.9)	0.292
	4B	13/40 (32.5)	6/42 (14.3)	15/41 (36.6)	7/33 (21.2)	
	40	12/14 (85.7)	20/45 (44.4)	11/28 (39.3)	18/32 (54.5)	
Objective	4A	4/46 (8.7)	2/26 (7.7)	1/13 (7.7)	4/36 (11.1)	0.473
(algorithm)	4B	14/37 (37.8)	12/48 (25.0)	13/54 (24.1)	13/42 (31.0)	
	40	9/12 (75.0)	13/21 (61.9)	13/28 (46.4)	9/16 (56.3)	

## DISCUSSION

The BI-RADS lexicon was created in order to standardize and promote uniformity in breast imaging reports. Although the BI-RADS Atlas provides examples of descriptors, there is variation among the radiologists who report them, in terms of the analysis and agreement^(9)^. Of the 199 lesions in our initial sample, 45 (22.6%) were found to be malignant. Of those 45 lesions, 27 (60.0%) showed nodular enhancement and 18 (40.0%) showed non-mass enhancement. This frequency is similar to that reported in previous studies, in which the prevalence of malignancy ranged from 22% to 55%^(10)^. Our most common result was invasive carcinoma of a nonspecific type.

The risk of malignancy for lesions classified as BI-RADS 4 is highly variable, ranging from 2% to 95%, and the PPV of biopsied lesions based on MRI findings ranges from 20% to 60%^(5-7)^. A meta-analysis of 18 articles collectively evaluating 2,556 lesions classified as BI-RADS 4 demonstrated that the risk of malignancy can vary from 2.5% to 18.3% for category 4A, from 23.5% to 57.1% for category 4B, and from 58.0% to 95.2% for category 4C^(11)^. Fujiwara et al.^(12)^ and Honda et al.^(13)^, respectively, recorded PPV values of 1.8% and 27.8% for category 4A; 11.8% and 79.2% for category 4B; and 67.5% and 98.4% for category 4C.

The BI-RADS reference values for category 4 subclassification range from 2% to 10% for 4A, 11% to 50% for 4B, and 51% to 96% for 4C. In our results, the subjective and objective analyses both demonstrated a tendency toward an increase in malignancy risk from subcategory 4A to subcategory 4C. However, the subjective analysis showed a discrepancy for category 4A, in which no malignant cases were identified, whereas the result of the objective analysis was closer to the reference limit (4.3%). For categories 4B and 4C, both approaches showed agreement with the literature, with the objective classification revealing greater proximity to the expected mean values.

Honda et al.^(13)^, analyzing 211 BI-RADS category 4 lesions (147 benign and 64 malignant), found malignancy in only one lesion in subcategory 4A, with a PPV of 1.8%. For subcategories 4B and 4C, the PPV values were 11.8% and 67.5%, respectively. The authors reported no significant differences in the distribution of lexical features between the subcategories, with the following exceptions^(13)^: for subcategory 4A-margins/internal enhancement of nodules, distribution of non-mass enhancement, and circumscribed margins/dark internal septations of nodules; for subcategories 4B and 4C-ring enhancement; and for subcategory 4C only-segmental distribution of non-mass enhancement. Similar results were presented by Strigel et al.^(14)^, who analyzed 82 category 4 breast lesions, showing PPVs for categories 4A, 4B, and 4C of 2.5%, 27.6%, and 83.3%, respectively.

By analyzing nodular enhancement and the probability of malignancy according to the significant features separately in the multivariate assessment, we were able to create an algorithm compatible with the known values for mammography and ultrasound. The algorithm can be used independently of the experience of the reporting expert, making it feasible to employ in clinical practice. Our results show that the algorithm proposed is applicable to nodules and can objectively assist in the interpretation of lesions seen on MRI, especially for those new to breast radiology. The subjective and objective analyses showed similar results for PPV. As detailed in the Results, validation of the proposed algorithm also indicated better agreement between radiologists in the subcategories in the objective analysis (using the algorithm) than in subjective analysis.

The subclassification of BI-RADS category 4 is not currently used for MRI, because of the scarcity of published data and the limited accuracy of the subcategories^(13)^. However, our results indicate an improvement in patient and physician expectations regarding the management of a result for which biopsy is indicated. Our findings also facilitate and support the anatomical-radiological correlation, which is mandatory in post-interventional procedure management.

Our study has some limitations. Such limitations include the retrospective design and the fact that it was a single-center study, as well as the small sample size, which particularly limited the analysis of non-mass lesions. In addition, the loss of images prevented consensus assessment, and the fact that T2-weighted and diffusion-weighted signals were not used for lesion classification limited the analysis. Furthermore, it should be noted that the study included all lesions initially classified as BI-RADS 4 on MRI, even if they presented likely benign imaging findings that could have been classified as BI-RADS 3. We believe that this portion of our sample was classified as BI-RADS 4 due to other factors that increase the risk of malignancy, such as new or enlarging lesions, high-risk patients or patients with known malignant tumors, and other criteria not directly related to imaging characteristics.

More than knowing which diagnostic investigation method to use for a BI-RADS 4 finding on MRI, it is important to understand the appropriate approach for findings visualized only on MRI. However, the stratification of the BI-RADS 4 category can help patients and physicians understand the risk of malignancy and make informed decisions about the appropriate course of action.

## CONCLUSION

The subcategorization of lesions into categories 4A, 4B, and 4C proved to be feasible, according to the risk of malignancy established by the BI-RADS lexicon criteria, both by the subjective analysis of experienced evaluators and by the objective analysis using the algorithm developed from the PPVs for the different descriptors employed.

The algorithm proposed, applicable to nodules, serves as a useful tool, especially for beginner breast radiologists or general practitioners who produce breast MRI reports. This tool provides greater confidence, ensuring a structured report and, in selected cases, enabling personalized follow-up with serial examinations instead of invasive procedures. Ultimately, this approach can improve the quality of patient care, providing care that is more individualized and effective.

## Data Availability

Datasets related to this article will be available upon request to the corresponding author.
